# An Unusual Case of Acute Foot Drop Caused by a Pseudoaneurysm

**DOI:** 10.1155/2011/515078

**Published:** 2011-07-31

**Authors:** Christopher J. Wong, Eric E. Kraus

**Affiliations:** ^1^Division of General Internal Medicine, Department of Medicine, University of Washington, P.O. Box 354760, 4245 Roosevelt Way NE, Seattle, WA 98105, USA; ^2^Department of Neurology, University of Washington, Seattle, WA 98105, USA

## Abstract

Lower extremity neurologic symptoms are a common presenting problem. Here we report the case of a 73-year-old man who developed acute right foot pain and foot drop. History, physical examination, and electrodiagnostic studies were consistent with a lumbosacral plexopathy. Imaging studies revealed an internal iliac artery pseudoaneurysm, a rare cause of acute foot drop.

## 1. Introduction

Lower extremity problems of the nervous system are a common complaint. For example, lumbosacral radiculopathy and polyneuropathy have prevalence rates of 3–5% and 3.6%, respectively [1, 2]. Localization of the lesion is a critical task in order to select appropriate diagnostic tests and treatment, and less common etiologies such as lumbosacral plexopathies are easily overlooked. Clinicians must maintain a high index of suspicion for vascular causes of acute lower extremity pain and weakness. In this case report we identify a patient with an acute lumbosacral plexopathy due to an internal iliac artery pseudoaneurysm.

## 2. Case Presentation

A 73-year-old man developed acute onset of right foot pain. The pain was sharp and burning in quality and located on the sole and dorsum of the right foot. He noted difficulty in walking, which he attributed to the pain. Four days later, he presented to the emergency department, concerned for a deep venous thrombosis. His past medical history included depression, atrial fibrillation for which he received warfarin therapy, hypertension, and prostate cancer treated 14 years prior by radical prostatectomy. 

On examination, the patient's temperature was 37.2 degrees Celsius, blood pressure 133/99 mm Hg, heart rate 82 beats per minute, respiratory rate 20 per minute, and oxygen saturation 95% on ambient air. He was alert and in no distress. Heart rhythm was irregularly irregular, without murmurs, rubs, or gallops. Examination of the lungs and abdomen was normal, and he had no edema. The dorsum and sole of the right foot were tender to palpation. Dorsalis pedis and posterior tibialis pulses were 2+ bilaterally. Strength was remarkable for 3/5 right ankle dorsiflexion, 4/5 right ankle plantar flexion, with the left lower extremity having 5/5 strength throughout. Reflexes were notable for 0/4 patellar and 0/4 Achilles reflexes on the right, compared with 2/4 patellar and 1/4 Achilles reflexes on the left. Biceps reflexes were 1/4 bilaterally. Sensation was normal to light touch. Upper extremity strength and reflexes were normal. 

**Figure 1:**
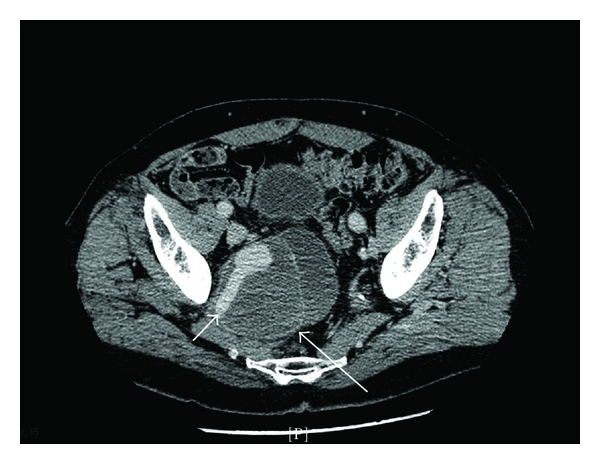
Right internal iliac artery pseudoaneurysm. A 7 × 9 cm hematoma (large arrow, posterior edge) arises from the right internal iliac artery with 2.5 × 3.5 cm saccular contrast opacification (small arrow).

**Figure 2:**
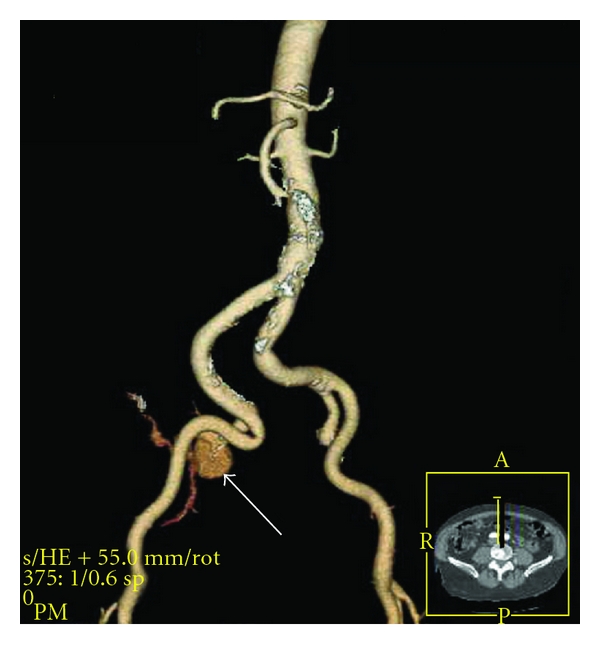
Reconstructed image of a pseudoaneurysm arising from the right internal iliac artery (arrow).

Laboratory studies showed a creatinine of 1.3 mg/dL (0.2–1.1), INR 1.8 (0.8–1.3), and otherwise normal serum chemistries, complete blood count, and prostate-specific antigen. Chest X-ray revealed clear lung fields and a tortuous aorta. A lumbar spine X-ray was normal. A lumbar spine MRI showed degenerative disc changes at L2/3. Electrodiagnostic studies revealed evidence of acute denervation of the right biceps femoris and tibialis anterior, and absent right peroneal motor nerve conduction to the extensor digitorum brevis. Paraspinal innervation was normal. A CT of the abdomen and pelvis with IV contrast discovered a 2.5 × 3.5 cm pseudoaneurysm of the right internal iliac artery and an associated 7.0 × 9.0 cm hematoma (Figures 1 and 2). 

The patient underwent coil embolization of the inflow and outflow arterial branches of the pseudoaneurysm. A stent graft was attempted but was unsuccessful. The patient's lower extremity pain resolved shortly after the embolization. Over the next 3 months, the patellar reflex returned but right foot dorsiflexion remained weak. 

## 3. Discussion

The causes of acute lower extremity symptoms include musculoskeletal, dermatologic, infectious, neoplastic, vascular, and neurologic disorders. In this case, despite the patient's initial concern for a deep venous thrombosis, his presentation was consistent with an acute neurologic problem, manifesting as painful foot drop. Identifying the cause of a lower extremity neurologic symptom such as foot drop can be challenging. A systematic approach can help elucidate the level of the neurologic lesion.

## 4. Making the Diagnosis: Localization

### 4.1. History and Physical Studies

Distal pain and weakness of one leg with reduced reflexes localize to the peripheral nervous system. He had no other signs to suggest a stroke or other central nervous system lesion. After deducing this presentation to be due to a peripheral nerve problem, the next step is to further localize along the course of the nerve. The possibilities from proximal to distal are nerve root, plexus, and peripheral nerve. This patient demonstrated an absent patellar reflex, absent Achilles reflex, weakness of ankle dorsiflexion, and pain of the dorsum and sole of the foot.  Table 1 illustrates the possible sites of involvement for each of these clinical findings, from nerve root to peripheral nerve. To explain the full set of findings, the following possibilities must be considered: a simultaneous process involving multiple nerve roots (L4-S1), a lumbosacral plexopathy, or, less likely, multiple concurrent sites of unilateral peripheral nerve disease (femoral nerve, and either sciatic nerve or both tibial and peroneal nerves). Multilevel nerve root disease and lumbosacral plexopathy affecting the lower extremities may be difficult to distinguish solely on the basis of history and exam. In some cases, the presence of pain or other symptoms may provide a diagnostic clue, but this patient did not have back, groin, or pelvic pain. 

### 4.2. Diagnostic Studies

Electrodiagnostic studies may assist in the localization of a peripheral nervous system problem and have higher specificity than sensitivity. Nerve root disease may show abnormal paraspinal innervation with preserved distal sensory nerve conduction because the lesion is proximal to the dorsal root ganglion. Plexopathies, on the other hand, may show normal paraspinal innervation but impaired sensory nerve conduction (e.g., sural nerve). This patient's electrodiagnostic studies suggested a lesion of, or proximal to, the sciatic nerve. Finally, imaging studies may assist in identifying a structural lesion to explain clinical findings and may include a spine MRI, CT, or MR-neurogram. This patient's spine MRI was negative for nerve root disease.

### 4.3. Putting History, Physical, and Diagnostic Studies Together

In some cases, no single finding makes the diagnosis, but all the evidence is required to localize the lesion. The history and physical studies suggested either multilevel nerve root disease, a plexopathy, or multiple affected peripheral nerves. The electrodiagnostic studies supported a lesion of, or proximal to, the sciatic nerve. However, the absent patellar reflex on exam additionally pointed to involvement of, or proximal to, the femoral nerve. Taken together with the normal spine MRI and normal paraspinal innervation, these findings should prompt consideration of a lumbosacral plexopathy. Significant causes of lumbosacral plexopathy include trauma, benign or malignant mass lesions, radiation injury, infiltrative disease, diabetes mellitus, and vascular disruption. If the cause of a lumbosacral plexopathy is not evident from the patient's history or exam, then imaging studies must be obtained. This patient's etiology was ultimately found to have been an acute injury to the nerves of the lumbosacral plexus due to a pseudoaneurysm of the internal iliac artery.

## 5. Internal Iliac Artery Aneurysm and Pseudoaneurysm

Internal iliac artery aneurysms are uncommon, with an estimated incidence of 2%, and those identified without other associated abdominal aneurysms are rare, with an estimated incidence of 0.4% [3]. Such aneurysms have been associated with severe constipation due to compression of the rectum [4], groin or abdominal pain, urinary retention, and hematuria [3]. Internal iliac artery aneurysms have been reported to present with chronic sciatic-type pain [5, 6], and common iliac artery aneurysms may also present with lumbosacral plexopathy and have been mistaken for progressive sciatic pain [7]. 

Lower extremity pain is an uncommon presentation of internal iliac artery aneurysms—in a review of 94 cases, only 13 reported lower extremity pain or weakness, of which nearly all were specified as being in the proximal lower extremity or had associated symptoms of back pain or abdominal pain [3]. This is the first case of which we are aware in which a patient presented with acute foot drop and foot pain as the only presenting complaint. Acute onset of lumbosacral plexopathy, as in the case of this patient, appears to be an uncommon presentation of a potentially serious vascular pseudoaneurysm. The suddenness of onset may have been a result of the expanding hematoma. 

Aneurysms of the internal iliac artery are critical to identify, as up to 40% may present with rupture [3]. Small, asymptomatic aneurysms may be observed with serial imaging. Larger aneurysms and those that are symptomatic may be treated with embolization and percutaneous placement of a vascular graft, as was the case with this patient. If embolization fails or is technically not possible, then open surgical repair is indicated. 

## Figures and Tables

**Table 1:** Clinical findings and anatomic correlates.

Clinical finding	Nerve root	Plexus	Peripheral nerve
Absent patellar reflex	L4	Lumbosacral plexus	Femoral
Weakness of ankle dorsiflexion	L5	Lumbosacral plexus	Sciatic or peroneal
Absent Achilles reflex	S1	Lumbosacral plexus	Sciatic or tibial
Pain on the dorsum and sole of foot	L5, S1	Lumbosacral plexus	Sciatic orperoneal + tibial
